# COVID-19 vaccination experience among United States dental professionals and students: Safety, confidence, concerns, and side effects

**DOI:** 10.1371/journal.pone.0264323

**Published:** 2022-02-22

**Authors:** Enas A. Bsoul, Peter M. Loomer

**Affiliations:** 1 Department of Comprehensive Dentistry, School of Dentistry, UT Health San Antonio, San Antonio, Texas, United States of America; 2 Dean of School of Dentistry, UT Health San Antonio, San Antonio, Texas, United States of America; Konkuk University, REPUBLIC OF KOREA

## Abstract

**Objectives:**

The purpose of this study was to evaluate the COVID-19 vaccination experience among United States-based dental professionals and students: to understand their beliefs, concerns, safety and confidence levels, and side effects experienced after vaccination; striving to boost vaccination acceptability to curtail the pandemic.

**Methods:**

An observational survey study approved by The University of Texas Health San Antonio Institutional Review Board was distributed to members of the School of Dentistry community using Qualtrics XM software. The survey was completed anonymously. Data were analyzed using R statistical computing software, χ^2^ test and Fisher’s Exact test.

**Results:**

Over 80% of all participants felt moderately to very safe working after the COVID-19 vaccine was made available, and more than 75% were moderately to very confident that the vaccine can protect them during the pandemic. At least 35% were moderately to very concerned about immediate and long-term side effects of the vaccine; despite the concerns, 94% received the vaccine. Side effects were more common after the second dose of the vaccine. Most common side effects were injection site pain, and general side effects of fatigue/tiredness, headache, muscle/body ache, and chills/fever. 74% reported no effect of the vaccine on daily activities, and the severity of side effects no worse than mild (about 60%).

**Conclusions:**

Majority of the participants felt safe and confident that the vaccine would protect them against COVID-19 infection. Sharing these findings and reliable information that the vaccine is safe and effective is paramount to fostering the vaccine uptake and curtailing the pandemic.

**Practical implications:**

Findings of this study demonstrated the confidence of the UT Health San Antonio, School of Dentistry community that the benefits of the vaccines greatly outweigh the risks; boosting the vaccination acceptance while creating a COVID-19 free environment both for the academic dental setting and the community it serves.

## Introduction

The severe acute respiratory syndrome coronavirus 2 (SARS-CoV-2) that causes the coronavirus disease-2019 (COVID-19) was first identified in Wuhan, China in December 2019 [1–3]. In late January 2020, the World Health Organization (WHO) declared a Public Health Emergency of International Concern, then declared a COVID-19 pandemic in March 2020 [1, 3, 4]. COVID-19 is one of the deadliest pandemics in history with more than 384 million cases confirmed, and 5.69 million deaths attributed to it as of February 3, 2022. COVID-19 variants of concern have emerged and become dominant in many countries since 2021, with Delta, Alpha and Beta being the most virulent variants. Omicron, the most recent variant of concern, has an immune escape ability that may allow it to spread via breakthrough infections, possibly allowing it to coexist with Delta, which more often infects the unvaccinated [3].

Highly variable symptoms of COVID-19 and incubation period of 7–24 days were reported; symptoms ranging from unnoticeable (flulike symptoms) to severe/life threatening [3, 5, 6]. According to Centers for Disease Control and Prevention (CDC), older adults and/or people with severe underlying medical conditions (such as heart or lung disease or diabetes) seem to be at higher risk for developing more serious complications [6]. Transmission of infection commonly occurs through exposure to respiratory droplets/small airborne particles, and via contaminated surfaces/fluids [3, 7–9].

Health professions were ranked at the highest risk for COVID-19 infection, with dentistry placed at the top [10–15]. CDC guidance recognized the unique nature of aerosol-generating dental procedures that warrant specific infection control considerations, and outlined preventive actions, included: getting a COVID-19 vaccine as soon as available, personal protective equipment, wearing masks, 6-feet social distancing, avoiding crowds, improving ventilation, and washing hands often [16].

On December 11, 2020, The United States (U.S.) Food and Drug Administration (FDA) issued the first emergency use authorization (EUA) for the Pfizer-BioNTech COVID-19 vaccine for individuals ≥16 years old, then expanded it to include adolescents ≥12 years old on May 10, 2021 [17, 18]. On August 23, 2021, FDA approved Comirnaty (COVID-19 Vaccine, mRNA), previously known as Pfizer-BioNTech, for the prevention of COVID-19 disease in individuals ≥16 years old.

Moderna COVID-19 vaccine was granted EUA on December 18, 2020 [19], followed by Johnson and Johnson’s/Janssen vaccine on February 27, 2021 [20]; both were for use in individuals ≥18 years old. COVID-19 vaccines can protect recipients from a SARS-CoV-2 infection by formation of antibodies and providing immunity against infection [21]. Pfizer and Moderna are mRNA vaccines, delivered in two shots. Janssen is a viral vector vaccine, delivered in a single shot. People are considered fully vaccinated 2 weeks after their second dose in a two dose vaccine, or 2 weeks after a single dose vaccine [22].

An ongoing COVID-19 vaccination campaign organized by CDC, with mass vaccinations in the U.S. began on December 14, 2020. By July 2021, the highly contagious Delta variant has caused a renewed surge in COVID-19 infections and became the predominant strain in the U.S. [23, 24]. Consequently, on July 27, 2021, CDC released an updated guidance to urgently increase vaccinations that play a crucial role in limiting virus spread and minimizing severe disease, with masking indoors in public places recommendation. COVID-19 vaccines authorized in the U.S. are highly effective at preventing severe disease, hospitalizations and death, including against the Delta variant [23].

On August 13, 2021, CDC recommended that moderately-to-severely immunocompromised people receive an additional dose of the COVID-19 vaccine. According to CDC’s data, as of February 2, 2022, approximately 64% of the country’s total population (over 212 million Americans) had been fully vaccinated, 75.4% had received at least one dose of the vaccine, and 41.8% (over 88.6 million Americans) had received a booster dose [25].

According to latest statistics, as of February 2, 2022, 59% of Texas population had been fully vaccinated, compared to 69.2% in California, 65.2% in Florida, and 74.3% in New York. Increasing numbers of COVID-19 cases were reported in the states with below-average vaccination rates, driving the surging spread of Delta variant, also increasing the chances of more concerning variants to emerge, making vaccination more urgent than ever [23, 24].

Side effects following vaccination might affect ability to do daily activities, most were mild-to-moderate, usually start within 1–2 days of getting the vaccine, and go away in a few days; they are normal signs that the body is building protection. CDC listed possible side effects to COVID-19 vaccines: localized pain/redness/swelling at injection site, general side effects of tiredness, muscle/joint pain, headache, chills/ever and nausea [17, 19, 20, 22]. General side effects were more common after the second dose of the Pfizer/Moderna vaccines [17, 19, 22].

Pfizer vaccine was reported to be 95% effective at preventing COVID-19 infection compared to 94.1% of Moderna and 66.3% of Janssen [22]. Less frequent side effects were reported to Pfizer compared to Moderna; however, Moderna is less temperature sensitive, easier to transport and store than Pfizer [21].

A U.S. study by Bartsch et al. [26], found that a COVID-19 vaccine of ≥70% efficacy is required to prevent an epidemic and of ≥80% to largely extinguish it, as the sole intervention. Reiter et al. [27], found that 69% of participants were willing to get a COVID-19 vaccine, highlighted the important role of healthcare providers and changeable health beliefs in vaccine trust and acceptability. Fisher et al. [28], found a concerning proportion of 42.2% hesitant to accept vaccination; black race reported as one of the strongest independent predictors of vaccine unacceptance. In addition, Solís Arce et al. [29], reported that vaccine acceptance was mainly explained by interest in personal protection against COVID-19 infection, concerns about vaccine side effects as the most common reason for hesitancy, and healthcare workers as the most trusted source of guidance regarding the vaccines. A large community-based study in U.S. found 22% of participants hesitant to take the COVID-19 vaccine if were available, with statistically significant differences based on sociodemographic characteristics; higher hesitancy found among African-Americans, Hispanics, those with lower education/incomes, with children at home, rural dwellers, northeastern U.S. people, and Republicans [30].

COVID-19 pandemic has become a race between effective vaccination and emerging variants. Various critical mutations in the spike protein of new variants would possibly make them more transmissible, infectious, and lethal. The possibility of more severe variants emerging in the near future, such as the Delta Plus variant-AY.1 (Delta sub-lineage) that had been declared by the Indian government as a variant of concern, is now becoming a new cause of global concern [31].

With the lack of specific therapy for COVID-19, widespread vaccination is a critical tool to help stop the pandemic. The purpose of this study was to evaluate the COVID-19 vaccination experience and side effects among U.S.-based dental professionals and students; findings can be utilized to counter misinformation, proactively send messages to foster the COVID-19 vaccine acceptance and potentially increase vaccination rates needed to reach herd immunity for curtailing the pandemic.

### Hypotheses

Hypotheses were that the majority of participants would feel safer and be confident in the protection afforded by the vaccine, but a good percentage would be at least moderately concerned about side effects. The majority would experience side effects no worse than mild, with no significant impact on their life activities. Differences were expected to be found among sex, race and age groups. The findings of this study were consistent with the hypotheses as discussed in the following sections.

## Materials and methods

An observational survey study that consisted of 17 items was approved by The University of Texas Health Science Center at San Antonio–UT Health San Antonio Institutional Review Board (Protocol Number: HSC20200374E), and distributed via an anonymous survey link to members of the UT Health San Antonio, School of Dentistry (SoD) using the web-based survey platform Qualtrics XM^™^ (Version January, 2021). Individuals who received the survey request had 47 days (January/29/2021-March/16/2021) to complete it and were sent three email reminders. The entire pool of students, staff and faculty at SoD were given an opportunity to receive a two-shot series of the Pfizer COVID-19 vaccine starting in mid-December/2020, then participate in a survey about the vaccine. Participants were asked questions about their beliefs regarding the vaccine, those who received at least one shot were also asked about their experience with side effects. The survey was completed anonymously with no incentive offered to participate.

### Statistical analysis

Data obtained from the survey responses were analyzed using R statistical computing software (R Core Team (2020)). R: A language and environment for statistical computing. R Foundation for Statistical Computing, Vienna, Austria). Statistical comparisons were done with the χ^2^ test, except Fisher’s Exact test was used when the number of participants in a category was too small to meet the requirements of the χ^2^ test. The reported *P* values are from the χ^2^ test. In addition to *P* values, 95% confidence intervals (CIs) for the proportions in each group were reported, along with odds ratios (ORs) comparing each group to the reference group. A level of significance of *P* equaling 0.05 was used for all statistical tests. Results of the survey were not weighted by response rate.

## Results

### Demographics

**Fig 1:**
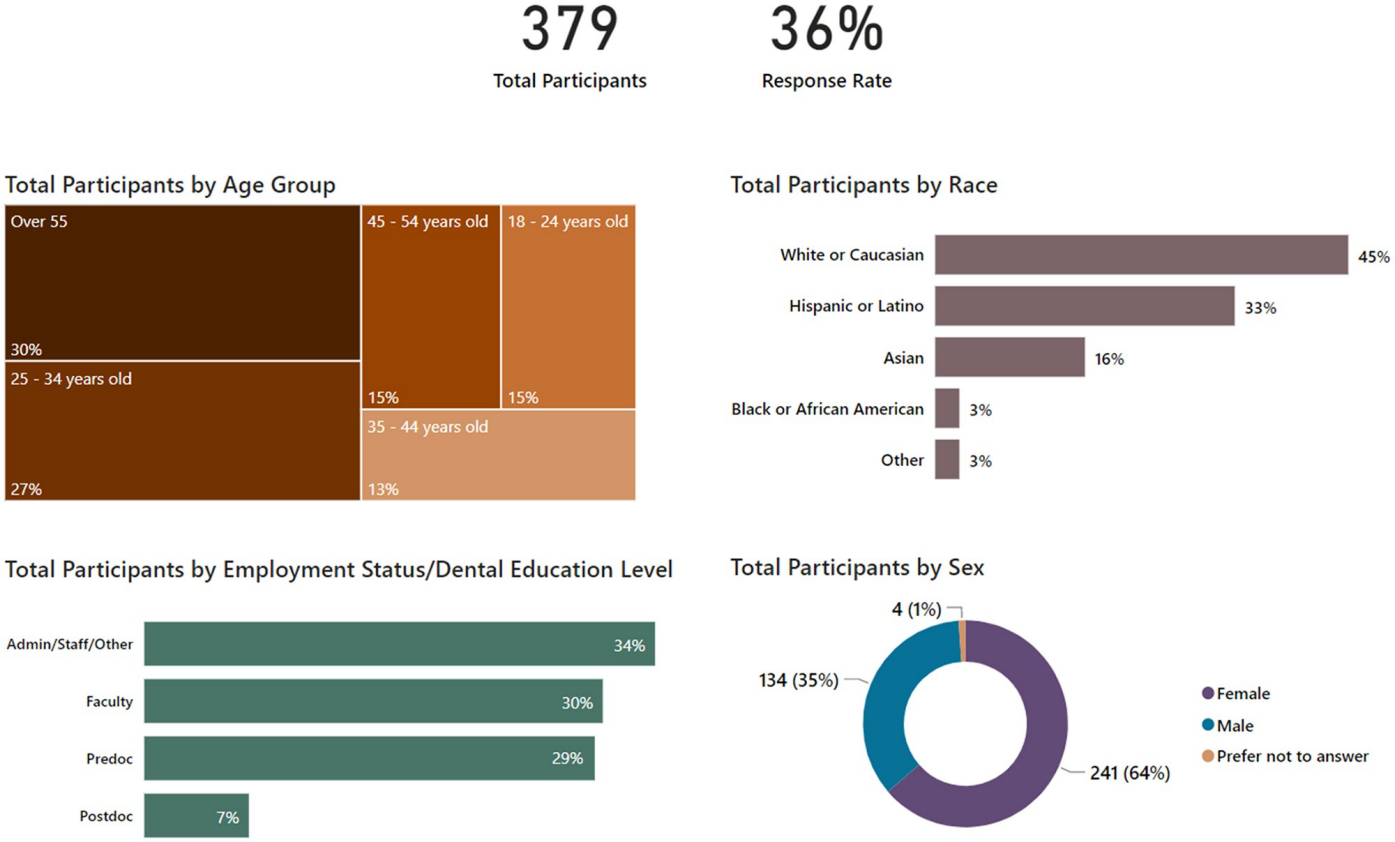
Demographics and groups distributions.

A total of 379 persons at SoD completed the first part of the survey, out of 1051 invited (36% response rate). 357 received at least one-shot by the time of the survey, all but one of them (99.7%) completed the second part of the survey. Response rates among various categories were: 60% of faculty, 43% of administrative/staff/other, 25% of predoctoral-students, and 22% of postdoctoral-students. Demographics are shown in Table 1 and Fig 1. Participants were generally representative of this particular SoD population, which is situated in a city with a high proportion of Hispanic residents but also attracts a fair number of international students. A total of 338 participants received both shots of the vaccine, and 19 received only the first shot at the time of taking the survey.

**Table 1:** Demographics and distribution of participants.

Characteristic		Completed Part 1	Completed Part 1 and 2
**Total Participants**		379	356
**Age**	18–24	56 (15%)	51 (14%)
	25–34	101 (27%)	95 (27%)
	35–44	51 (13%)	49 (14%)
	45–54	58 (15%)	53 (15%)
	55 or older	113 (30%)	108 (30%)
**Sex**	Female	241 (64%)	227 (64%)
	Male	134 (35%)	127 (36%)
	Preferred not to Answer	4 (1%)	2 (1%)
**Race**	Asian	62 (16%)	61 (17%)
	Black	10 (3%)	8 (2%)
	Hispanic	124 (33%)	119 (33%)
	Other	12 (3%)	11 (3%)
	White	171 (45%)	157 (44%)
**Education**	H.S./Some college	62 (16%)	57 (16%)
	Assoc. or Bach. Degree	111 (29%)	100 (28%)
	Master’s or Ph.D.	67 (18%)	64 (18%)
	DDS/DMD	124 (33%)	121 (34%)
	Other Professional Degree	15 (4%)	14 (4%)
**Status at School**	Dental Hygiene Student	13 (3%)	10 (3%)
	Dental Student, Yr 1 to 4	99 (26%)	93 (26%)
	Advanced Studies	26 (7%)	24 (7%)
	Faculty	114 (30%)	111 (31%)
	Admin/Staff/Other	127 (34%)	118 (33%)
**Covid Positive in Past?**	No	354 (93%)	340 (96%)
	Yes	25 (7%)	16 (4%)

### Beliefs about the vaccine

**Fig 2:**
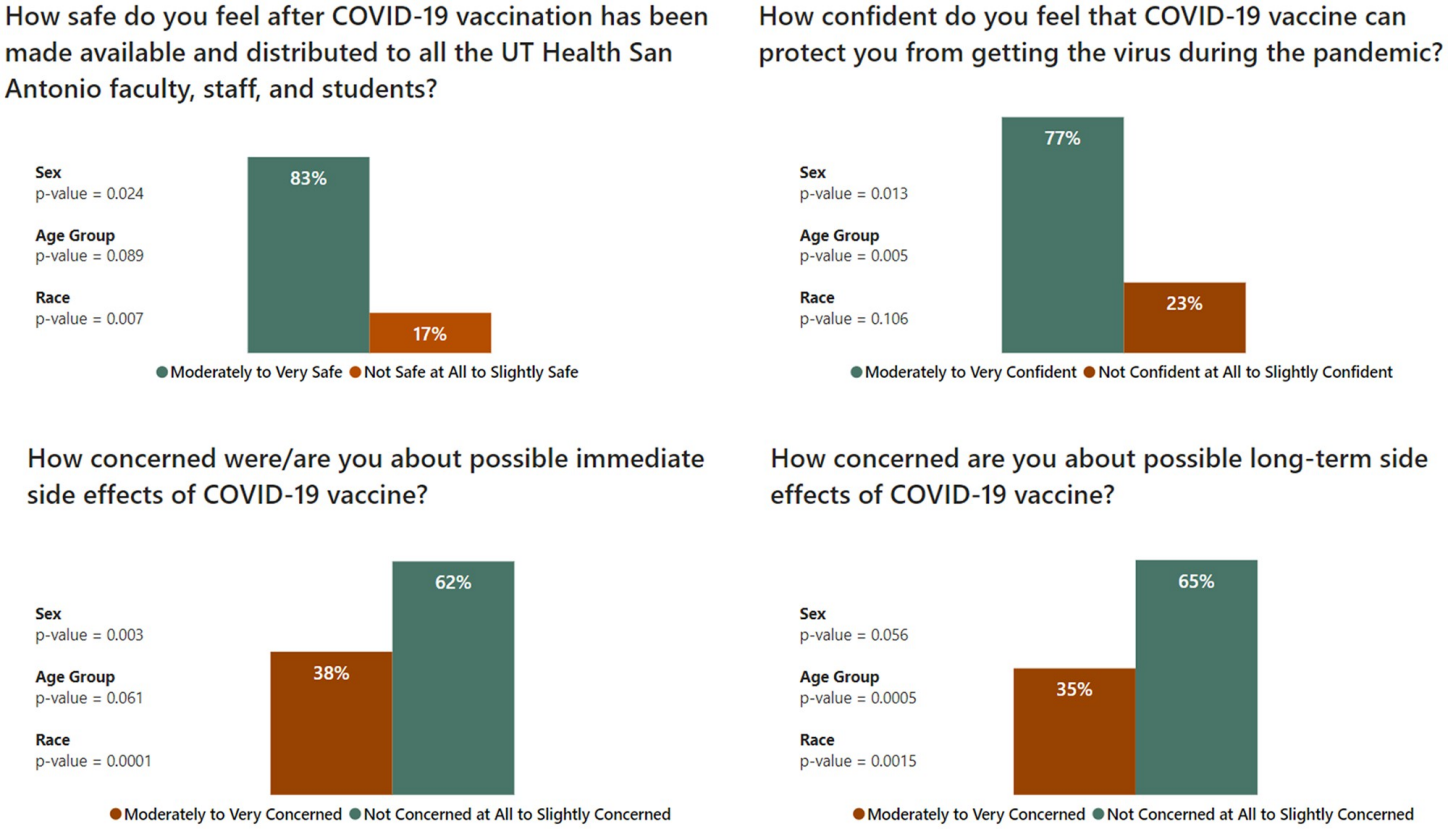
Beliefs/Opinions for all participants (*P* value by sex, age group and race).

Participants in the first part were asked to provide their belief/opinion on a 5-point scale on four statements about confidence in the vaccine and concerns about side effects. Using a standard methodology for surveys, their answers were grouped into two categories to reflect either an optimistic or a neutral/negative outlook (moderately-to-very vs. not-to-slightly) as shown in Fig 2. Age groups were combined into three groups (18–34, 35–54, 55+) for comparisons. Statistical comparisons of responses were done by the following demographic groups: Sex, age group, and race/ethnicity.

At least 35% of all participants were moderately-to-very concerned about both immediate and long-term side effects of the vaccine. Men, Whites, and oldest age group (55+) were less likely to be concerned. Among males, 28% (95% CI of 20%-35%) were moderately-to-very concerned about immediate side effects compared to 43% of females (95% CI of 37%-50%), OR = 0.50, p-value = 0.003, whereas 26% of whites (CI:20%-33%) were moderately-to-very concerned about immediate side effects compared to 45% of Asians (CI:33%-57%), OR = 0.43, and 49% of Hispanics (CI:40%-58%), OR = 0.37, p = 0.0001 for race. When asked about long-term side effects, 24% of whites (CI:18%-30%) were moderately-to-very concerned, compared to 40% of Asians (CI:28%-53%), OR = 0.47, and 43% of Hispanics (CI:34%-51%), OR = 0.42, p = 0.002 for race, while 21% of participants aged 55+ (CI:14%-29%) were moderately-to-very concerned compared to 46% of the 35–54 year group (CI:36%-55%), OR = 0.32, and 37% of the 18–34 year group (CI:29%-44%), OR = 0.46, p = 0.0005 for age group. Despite the concerns about side effects, a very high percentage of the participants (94%) took the vaccine.

Over 75% of participants were moderately-to-very confident that the vaccine can protect them from getting the virus. Women and those aged 18–54 were less confident on average about the protection offered by the vaccine. For women, 73% (CI:69%-79%) were moderately-to-very confident, compared to 84% of men (CI:78%-90%, OR = 0.50, p = 0.013). On the other hand, 72% (CI:67%-77%) of the younger two groups were moderately-to-very confident, compared to 88% of those 55+ (CI:82%-94%, OR = 0.37, p = 0.005). >80% of all participants felt moderately-to-very safe working at SoD after the vaccine was made available. Men and Whites were more likely to feel safest. Among men, 89% (CI:84%-94%) felt moderately-to-very safe, compared to 80% of women (CI:75%-85%, OR = 2.02, p = 0.024), while 89% of whites (CI:84%-94%) felt moderately-to-very safe, compared to 82% of Asians (CI:72%-92%, OR = 1.83), and 76% of Hispanics (CI:68%-84%, OR = 2.71), p = 0.007 for race.

### Report of side effects and impact on life activities

**Fig 3:**
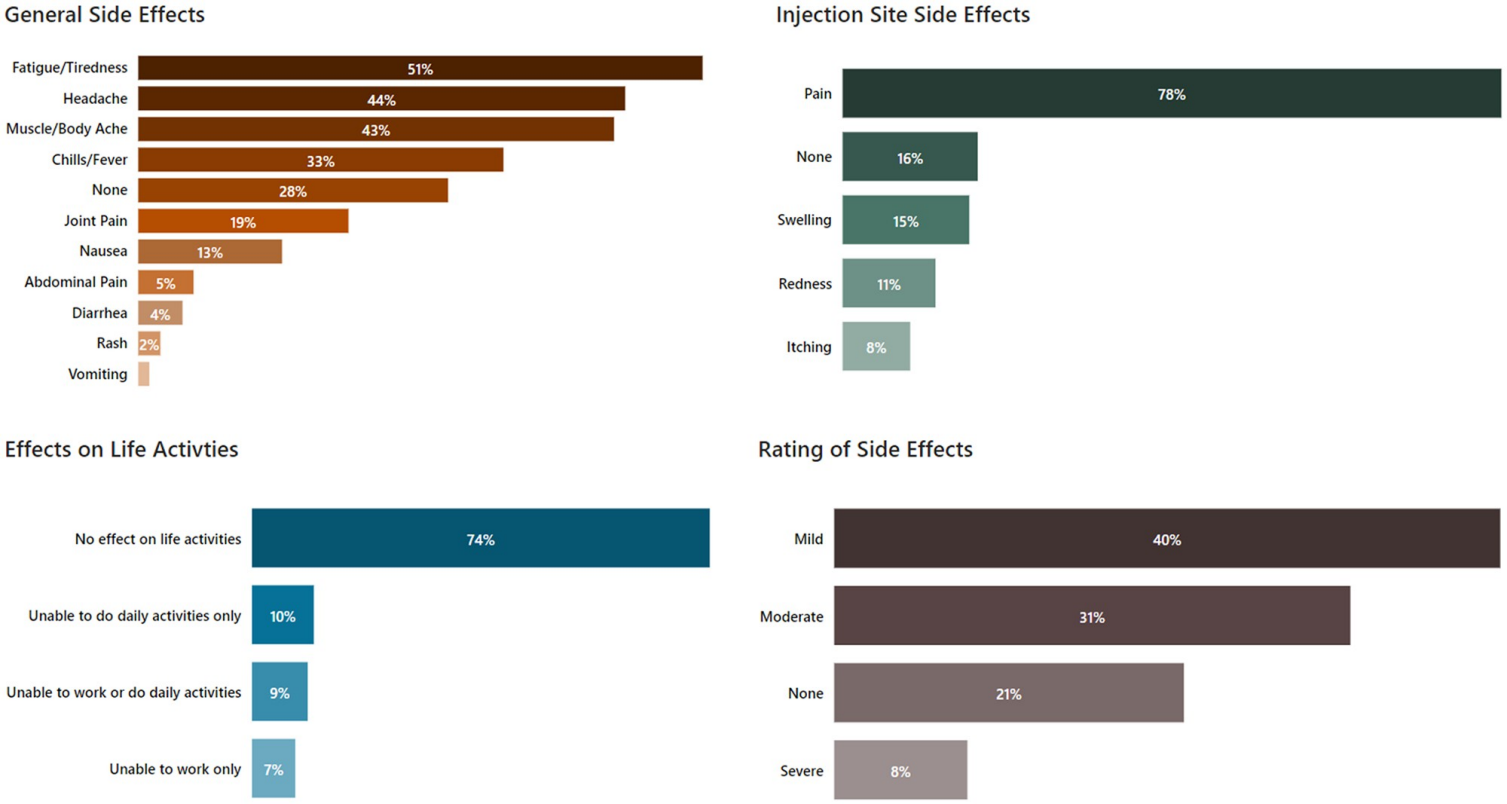
Side effects, rating of severity, and effects on life activities for all participants.

**Fig 4:**
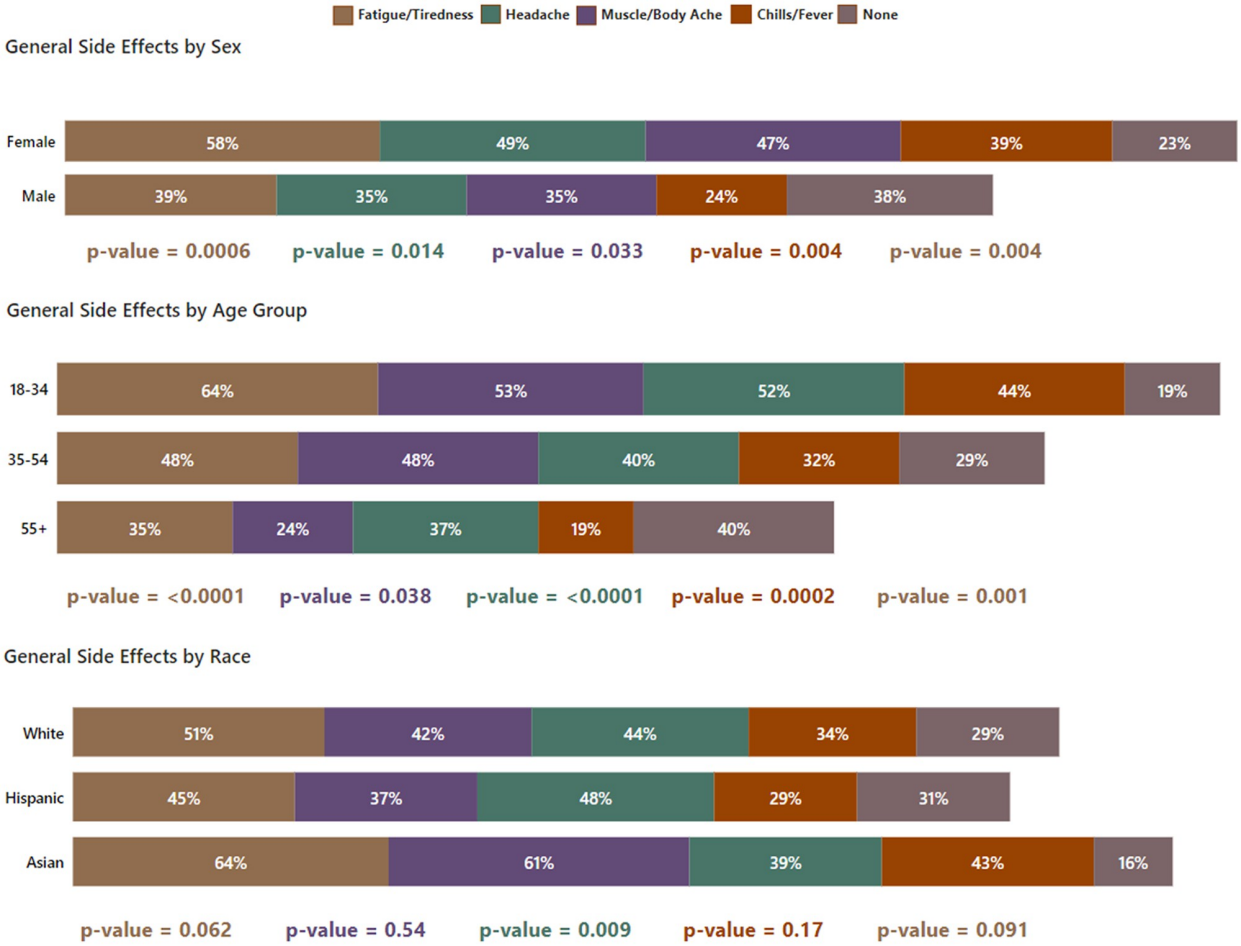
General side effects by sex, age group and race.

The participants who completed the second part were asked to document injection site and general side effects they experienced in the first few days after receiving their shots, to rate the severity of the side effects, and how the side effects affected their ability to work/do daily activities. Responses on side effects, severity and impact on life activities are shown in Fig 3. General side effects by sex, age group and race are shown in Fig 4. Responses about symptoms/side effects were rated independently because it was anticipated that some participants would experience more than one symptom. Only one participant reported a need to get care from a doctor for their vaccine side effects.

The most common side effects were injection site pain (in 3 out of 4 participants), and the following general side effects (in ⅓-½ of participants): fatigue/tiredness, headache, muscle/body ache, and chills/fever. One in six participants noted no injection site side effects, and one in four reported no general side effects. About 60% of participants reported the severity of side effects to be no worse than mild, and almost three-quarters stated the vaccine did not interfere with their life activities. Side effects after both doses of the vaccine were reported by 36% of all participants, after the second dose only by 27%, after the first dose only by 14%, while 23% of the participants didn’t experience any side effects.

Women were more likely to experience moderate-to-severe side effects (46% of women, CI:40%-52%, compared to 29% of men, CI:21%-37%, OR = 2.06, p = 0.006), were also more likely to experience an impact on life activities (31% of women, CI:25%-37%, compared to 17% of men, CI:10%-24%, OR = 2.17, p = 0.014). The only statistically significant difference in injection site side effects between men and women was more swelling among women (18% of women, CI:13%-23%, compared to 9% of men, CI:4%-14%, OR = 2.32, p = 0.017). Men were more likely to experience no general side effects (38% of men, CI:29%-47%, compared to 23% of women, CI:17%-29%, OR = 1.99, p = 0.004), and women were more likely to experience fatigue/tiredness (58% of women, CI:52%-64%, compared to 39% of men, CI:30%-48%, OR = 2.17, p = 0.0006), headache (49% of women, CI:43%-55%, compared to 35% of men, CI:27%-43%, OR = 1.74, p = 0.014), muscle/body ache (47% of women, CI:40%-54%, compared to 35% of men, CI:27%-43%, OR = 1.62, p = 0.033), chills/fever (39% of women, CI:33%-45%, compared to 24% of men, CI:17%-31%, OR = 2.05, p = 0.004), nausea (16% of women, CI:11%-21%, compared to 8% of men, CI:3%-13%, OR = 2.28, p = 0.025), and abdominal pain (7% of women, CI:4%-10%, compared to 2% of men, CI:0%-4%, OR = 4.74, p = 0.025).

Participants under age 55 were more likely to experience moderate-to-severe side effects than the oldest group, with 48% (CI:40%-56%, OR = 2.76)) of the youngest group and 43% (CI:33%-53%, OR = 2.28) of the middle group, compared to 25% of the 55+ group (CI:17%-33%), p = 0.002. Participants under age 55 were also more likely to experience impact on life activities compared to the oldest participants, with 30% of the youngest group (CI:23%-37%, OR = 2.40), and 33% of the middle group (CI:24%-42%, OR = 2.88) compared to 15% of the oldest group (CI:8%-22%), p = 0.041. The oldest group was less likely to experience injection site pain, with 65% (CI:56%-74%) compared to 87% of the middle group (CI:80%-94%,OR = 0.27) and 82% of the youngest group (CI:76%-88%, OR = 0.40), p = 0.0001, oldest group was also more likely to experience no general side effects, with 40% (CI:31%-49%) compared to 29% of the middle group (CI:20%-38%, OR = 1.59) and 19% of the youngest group (CI:13%-25%, OR = 2.79), p = 0.001. There were also age group-related differences in fatigue/tiredness, headache, muscle/body ache and chills/fever, with the youngest group experiencing those side effects most often and the oldest group experiencing them least often. The rate of fatigue/tiredness in the youngest group was 64% (CI:56%-72%) compared to 48% of the middle group (CI:38%-58%, OR = 1.96) and 35% of the 55+ group (CI:26%-44%, OR = 3.33), p<0.0001. The rate of headache in the youngest group was 52% (CI:44%-60%) compared to 40% of the middle group (CI:30%-50%, OR = 1.62) and 37% of the oldest group (CI:28%-46%, OR = 1.85), p = 0.038. The rate of muscle/body ache in the youngest group was 53% (CI:45%-61%) compared to 48% of the middle group (CI:38%-58%, OR = 1.21) and 24% of the 55+ group (CI:16%-32%, OR = 3.52), p<0.0001. Finally, the rate of chills/fever in the youngest group was 44% (CI:36%-52%) compared to 32% of the middle group (CI:23%-41%, OR = 1.63) and 21% of the oldest group (CI:13%-29%, OR = 3.23), p = 0.0002.

There were no statistically significant differences in severity or impact on life activities among the three largest race groups (White, Hispanic and Asian). There were only a few differences in specific side effects by race, with more Hispanics experiencing injection site swelling (23%, CI:15%-31%) compared to 10% of Whites (CI:5%-15%, OR = 2.59) and 11% of Asians (CI:3%-19%, OR = 2.26), p = 0.011 for this symptom, and Asians experiencing more muscle/body ache (61%, CI:49%-73%) compared to 42% of Whites (CI:34%-50%, OR = 2.13) and 37% of Hispanics (CI:28%-46%, OR = 2.63), p = 0.009.

Participants who received at least one shot were offered a chance to comment about their experience using an open-ended question. Of the 105 participants who provided comments, the most common responses were praise on the efficiency of the scheduling/on-site process (48 participants), appreciation for (20) and pride at (11) receiving the vaccine so early. However, 3 experienced wait times on site of over an hour, 5 had suggestions for improving the administration process, and 8 expressed concerns about policies/choices by others. Finally, 9 persons reported other side effects than those listed on the survey, including one with a serious allergic reaction and two with increases in their blood glucose levels.

## Discussion

Profession of dentistry has been ranked amongst the highest risk for COVID-19 infection [10–15]. CDC guidelines stressed receiving a COVID-19 vaccine as a crucial preventive measure to protect against COVID-19 infection and the fast spreading variants [9]. Healthcare providers, were among the first to receive the COVID-19 vaccine. This study has shed light on the beliefs/concerns/safety and confidence of dental professionals and students regarding the vaccine, and investigated the symptoms/side effects they experienced. The positive findings of this study may contribute to alleviate stress/anxiety, increase confidence, and reassure individuals who are hesitant to receive the vaccine. Vaccination hesitancy may be reduced given credible information that a vaccine is safe, effective, and with few side effects [28].

The number/proportion of participants by demographic category in this study were very similar to those in previous survey studies conducted during the pandemic on COVID-19 impact and challenges [14, 15].

Studies conducted in U.S. found participants to be more willing to get vaccinated if it would be recommended by their healthcare provider [27, 29], also found COVID-19 vaccine hesitancy differences to be based on/may vary by some sociodemographic characteristics such as sex, ethnicity/race, income, education, employment status, and residence place [27, 28, 30]. Political affiliation and perceived threat of COVID-19 were also reported as strong predictors of vaccine hesitancy [30]. Our study found >80% of all participants felt moderately-to-very safe working at SoD after the vaccine was made available; men and Whites were more likely to feel safest. >75% were moderately-to-very confident that the vaccine can protect them; women and those aged 18–54 were less confident on average. ≥35% were moderately-to-very concerned about immediate and long-term side effects; men, Whites, and oldest age group were less likely to be concerned. Despite the concerns, 94% took the vaccine, rates that were higher than the feeling of safety and confidence level expressed by participants.

General side effects were more common after the second dose of the Pfizer vaccine, and the most common side effects reported in this study were in agreement with those listed by CDC and FDA [17, 19, 20, 22]. Women and participants under age 55 were more likely to experience moderate-to-severe side effects and an impact on life activities. The majority of participants reported the vaccine had no effect on their life activities and severity of side effects no worse than mild; very important findings to boost COVID-19 vaccination, since concerns about vaccine side effects were reported as the most common reason for vaccine hesitancy [29].

Our findings are consistent with the hypotheses; the majority of participants felt safer, were confident in the protection afforded by the vaccine despite being concerned about side effects, and the majority experienced side effects no worse than mild, with no significant impact on their life activities. In addition, some differences were highlighted among sex, race and age groups.

### Practical implications

Our findings demonstrated the confidence of the SoD community that the benefits of the vaccines greatly outweigh the risks; boosting the vaccination acceptance while creating a COVID-19 free environment for academic dental setting and the community it serves.

### Limitations

This study was conducted amongst a dental school population, who were offered only one type of vaccine. The demographics may not mimic of dental school populations in other parts of the country. Further, individuals’ experiences with other types of vaccines may differ from the one used in this study. Future, nation-wide, larger sample size research, inclusive of a more diverse population, and evaluating possible long-term side effects of various COVID-19 vaccines is needed.

## Conclusions

The majority of dental professionals and students felt moderately-to-very safe working at SoD after the COVID-19 vaccine was made available, and were moderately-to-very confident that the vaccine can protect them from getting the virus. At least 35% were moderately-to-very concerned about immediate and long-term side effects; despite the concerns, 94% took the vaccine.

The majority reported no effect of the vaccine on their life activities and the severity of side effects no worse than mild. Women and participants under age 55 were more likely to experience moderate-to-severe side effects and an impact on life activities.

## Supporting information

S1 File Survey document. (DOCX)

## References

[1] Sohrabi C, Alsafi Z, O’Neill N, Khan M, Kerwan A, Al-Jabir A, et al. World Health Organization declares global emergency: a review of the 2019 novel coronavirus (COVID-19). Int J Surg. 2020;76:71–76. doi: 10.1016/j.ijsu.2020.02.034 32112977 PMC7105032

[2] Lu H, Stratton CW, Tang Y-W. Outbreak of pneumonia of unknown etiology in Wuhan, China: the mystery and the miracle. J Med Virol. 2020;92(4):401–402. doi: 10.1002/jmv.25678 31950516 PMC7166628

[3] Wikipedia [Internet]. COVID-19 pandemic; 2022 [updated 2022 February 3; cited 2022 February 3]. https://en.wikipedia.org/wiki/COVID-19_pandemic

[4] Cascella M, Rajnik M, Cuomo A, Dulebohn SC, Di Napoli R. Features, evaluation and treatment Coronavirus (COVID-19). StatPearls. 2020. StatPearls Publishing. 32150360

[5] Chen N, Zhou M, Dong X, Qu J, Gong F, Han Y, et al. Epidemiological and clinical characteristics of 99 cases of 2019 novel coronavirus pneumonia in Wuhan, China: a descriptive study. Lancet. 2020;395(10223):507–513. doi: 10.1016/S0140-6736(20)30211-7 32007143 PMC7135076

[6] Centers for Disease Control and Prevention [CDC]. Symptoms of COVID-19; 2021 [updated 2021 February 22; cited 2021 August 16]. https://www.cdc.gov/coronavirus/2019-ncov/symptoms-testing/symptoms.html

[7] Kutter JS, Spronken MI, Fraaij PL, Fouchier RA, Herfst S. Transmission routes of respiratory viruses among humans. Curr Opin Virol. 2018;28:142–151. doi: 10.1016/j.coviro.2018.01.001 29452994 PMC7102683

[8] Ather A, Patel B, Ruparel NB, Diogenes A, Hargreaves KM. Coronavirus disease 19 (COVID-19): implications for clinical dental care. J Endod. 2020;46(5):584–595. doi: 10.1016/j.joen.2020.03.008 32273156 PMC7270628

[9] Centers for Disease Control and Prevention [CDC]. How COVID-19 spreads; 2021 [updated 2021 July 14; cited 2021 August 16]. https://www.cdc.gov/coronavirus/2019-ncov/prevent-getting-sick/how-covid-spreads.html

[10] Al Kawas S, Al-Rawi N, Talaat W, Hamdoon Z, Salman B, Al Bayatti S, et al. Post COVID-19 lockdown: measures and practices for dental institutes. BMC Oral Health. 2020;20(1):291. doi: 10.1186/s12903-020-01281-6 33109185 PMC7590562

[11] Barabari P, Moharamzadeh K. Novel coronavirus (COVID-19) and dentistry-a comprehensive review of literature. Dent J (Basel). 2020;8(2):53. 10.3390/dj8020053 PMC7345990 32455612

[12] L. Gamio. The workers who face the greatest coronavirus risk. *New York Times*; 2020 April 2. https://www.nytimes.com/interactive/2020/03/15/business/economy/coronavirus-worker-risk.html

[13] Ahmadi H, Ebrahimi A, Ghorbani F. The impact of COVID-19 pandemic on dental practice in Iran: a questionnaire-based report. BMC Oral Health. 2020;20(1):354. doi: 10.1186/s12903-020-01341-x 33272261 PMC7711254

[14] Bsoul EA, Loomer PM. Mitigating the impact of COVID-19 on dental education and the resumption of patient care: The UT Health San Antonio experience. J Interdis Clin Dent. 2021;2(5):1–11.

[15] Bsoul EA, Challa SN, Loomer PM. Multifaceted impact of COVID-19 on dental practice: American dental care professionals prepared and ready during unprecedented challenges. J Am Dent Assoc. 2022;153(2):132–143. doi: 10.1016/j.adaj.2021.07.023 34763816 PMC8520822

[16] Centers for Disease Control and Prevention [CDC]. Guidance for dental settings. Interim infection prevention and control guidance for dental settings during the coronavirus disease 2019 (COVID-19) pandemic; 2020 [updated 2020 December 4; cited 2021 August 16]. https://www.cdc.gov/coronavirus/2019-ncov/hcp/dental-settings.html

[17] U.S. Food and Drug Administration [FDA]. Comirnaty and Pfizer-BioNTech COVID-19 Vaccine; 2022 [updated 2022 February 1; cited 2022 February 3]. https://www.fda.gov/emergency-preparedness-and-response/coronavirus-disease-2019-covid-19/comirnaty-and-pfizer-biontech-covid-19-vaccine

[18] U.S. Food and Drug Administration [FDA]. Pfizer-BioNTech COVID-19 Vaccine EUA Letter of Authorization reissued 05-10-2021; 2021 [updated 2021 May 10; cited 2021 August 16]. https://www.fda.gov/media/144412/download

[19] U.S. Food and Drug Administration [FDA]. Spikevax and Moderna COVID-19 Vaccine; 2022 [updated 2022 February 1; cited 2022 February 3]. Spikevax and Moderna COVID-19 Vaccine | FDA

[20] U.S. Food and Drug Administration [FDA]. Janssen COVID-19 Vaccine; 2022 [updated 2022 February 1; cited 2022 February 3]. https://www.fda.gov/emergency-preparedness-and-response/coronavirus-disease-2019-covid-19/janssen-covid-19-vaccine

[21] Meo SA, Bukhari IA, Akram J, Meo AS, Klonoff DC. COVID-19 vaccines: comparison of biological, pharmacological characteristics and adverse effects of Pfizer/BioNTech and Moderna Vaccines. Eur Rev Med Pharmacol Sci. 2021;25(3):1663–1669. doi: 10.26355/eurrev_202102_24877 33629336

[22] Centers for Disease Control and Prevention [CDC]. Different COVID-19 vaccines; 2022 [updated 2022 January 21; cited 2022 February 3]. https://www.cdc.gov/coronavirus/2019-ncov/vaccines/different-vaccines.html

[23] Centers for Disease Control and Prevention [CDC]. Delta variant: What we know about the science; 2021 [updated 2021 August 26; cited 2021 September 7]. https://www.cdc.gov/coronavirus/2019-ncov/variants/delta-variant.html

[24] Wikipedia [Internet]. COVID-19 vaccination in the United States; 2022 [updated 2022 February 2; cited 2022 February 3]. https://en.wikipedia.org/wiki/COVID-19_vaccination_in_the_United_States

[25] Centers for Disease Control and Prevention [CDC]. COVID Data Tracker. COVID-19 vaccinations in the United States; 2022 [updated 2022 February 2; cited 2022 February 3]. https://covid.cdc.gov/covid-data-tracker/#vaccinations

[26] Bartsch SM, O’Shea KJ, Ferguson MC, Bottazzi ME, Wedlock PT, Strych U, et al. Vaccine efficacy needed for a COVID-19 coronavirus vaccine to prevent or stop an epidemic as the sole intervention. Am J Prev Med. 2020;59(4):493–503. doi: 10.1016/j.amepre.2020.06.011 32778354 PMC7361120

[27] Reiter PL, Pennell ML, Katz ML. Acceptability of a COVID-19 vaccine among adults in the United States: How many people would get vaccinated? Vaccine. 2020;38(42):6500–6507. Epub 2020 Aug 20. doi: 10.1016/j.vaccine.2020.08.043 . 32863069 PMC7440153

[28] Fisher KA, Bloomstone SJ, Walder J, Crawford S, Fouayzi H, Mazor KM. Attitudes toward a potential SARS-CoV-2 vaccine: a survey of U.S. adults. Ann Intern Med. 2020;173(12):964–973. Epub 2020 Sep 4. doi: 10.7326/M20-3569 . 32886525 PMC7505019

[29] Solís Arce JS, Warren SS, Meriggi NF, Scacco A, McMurry N, Voors M, et al. COVID-19 vaccine acceptance and hesitancy in low- and middle-income countries. Nat Med. 2021;27(8):1385–1394. Epub 2021 Jul 16. doi: 10.1038/s41591-021-01454-y . 34272499 PMC8363502

[30] Khubchandani J, Sharma S, Price JH, Wiblishauser MJ, Sharma M, Webb FJ. COVID-19 vaccination hesitancy in the United States: a rapid national assessment. J Community Health. 2021;46(2):270–277. Epub 2021 Jan 3. doi: 10.1007/s10900-020-00958-x . 33389421 PMC7778842

[31] Thakur V, Bhola S, Thakur P, Patel SKS, Kulshrestha S, Ratho RK, et al. Waves and variants of SARS-CoV-2: understanding the causes and effect of the COVID-19 catastrophe [published online ahead of print, 2021 Dec 16] [published correction appears in Infection. 2022 Jan 14;:]. Infection. 2021;1–16. 10.1007/s15010-021-01734-2 PMC8675301 34914036

