# Supplementary material for: COVID-19 vaccination experience among United States dental professionals and students: Safety, confidence, concerns, and side effects
Source: PLoS One. 2022 Feb 22;17(2):e0264323. doi: 10.1371/journal.pone.0264323 (PMC8863258; doi:10.1371/journal.pone.0264323)
Supplement: Supplementary file 1 [file pone.0264323.s001.docx]

COVID-19 Vaccination Experience at UT Health San Antonio Dentistry

Start of Block: Demographics

Q1 What is your current employment status or dental education level at UT Health San Antonio Dentistry?

- Faculty 

- Administrative/staff 

- Dental student 

- Dental hygiene student 

- Graduate/postgraduate student 

- Other 

Q2 What is your age?

- Under 18 

- 18 - 24 years old 

- 25 - 34 years old 

- 35 - 44 years old 

- 45 - 54 years old 

- Over 55 

Q3 What is your sex?

- Male 

- Female 

- Other 

- Prefer not to answer 

Q4 What is your race/ethnicity?

- White or Caucasian 

- Hispanic or Latino 

- Black or African American 

- American Indian or Alaska Native 

- Asian 

- Native Hawaiian or Pacific Islander 

- Other 

Q5 What is your educational level?

- Less than high school 

- High school graduate 

- Some college 

- Bachelor's or Associate degree 

- Master's degree 

- Professional degree DDS/DMD 

- Professional degree DH 

- Professional degree/other 

- PhD 

End of Block: Demographics

Start of Block: COVID-19 Vaccine

Q6 Have you had a positive COVID-19 test or were told by a health care provider that you had COVID-19? 

- Yes 

- No 

Q7 How safe do you feel after COVID-19 vaccination has been made available and distributed to all the UT Health San Antonio faculty, staff and students?

- Very safe 

- Moderately safe 

- Slightly safe 

- Not so safe 

- Not safe at all 

Q8 How confident do you feel that COVID-19 vaccine can protect you from getting the virus during the pandemic?

- Very confident 

- Moderately confident 

- Slightly confident 

- Not so confident 

- Not confident at all 

Q9 How concerned were/are you about possible immediate side effects of COVID-19 vaccine?

- Very concerned 

- Moderately concerned 

- Slightly concerned 

- Not so concerned 

- Not concerned at all 

Q10 How concerned are you about possible long-term side effects of COVID-19 vaccine?

- Very concerned 

- Moderately concerned 

- Slightly concerned 

- Not so concerned 

- Not concerned at all 

Q11 Have you received your COVID-19 vaccine?

- Yes, first dose only 

- Yes, first and second doses 

- No 

Skip To: End of Survey If Have you received your COVID-19 vaccine? = No

End of Block: COVID-19 Vaccine

Start of Block: Symptoms Check and Health Impact

Q12 Regarding your COVID-19 vaccination experience, did you experience any symptoms after vaccination?

- Yes, after the first dose only 

- Yes, after the second dose only 

- Yes, after both doses 

- I didn't experience any symptoms 

Q13 Have you had any of these symptoms at the injection site (where you got the shot)? 
Select all that apply.

- Pain 

- Redness 

- Swelling 

- Itching 

- None 

Q14 Have you experienced any of these symptoms?
Select all that apply.

- Chills/fever 

- Headache 

- Joint pains 

- Muscle or body aches 

- Fatigue or tiredness 

- Nausea 

- Vomiting 

- Diarrhea 

- Abdominal pain 

- Rash (not including the immediate area around the injection site) 

- None 

Q15 If you experienced any symptoms, how would you rate them?

- Mild: you notice symptoms, but they aren't a problem 

- Moderate: symptoms that limit your normal daily activities 

- Severe: symptoms make normal daily activities difficult or impossible 

- I experienced no symptoms 

Q16 Did any of your vaccine-related symptoms or health conditions cause you to: 
Select all that apply.

- Be unable to work 

- Be unable to do your normal daily activities 

- Get care from a doctor or other health care professional 

- None of the above 

Q17 Do you have any other comments regarding your COVID-19 vaccination experience?

________________________________________________________________

________________________________________________________________

________________________________________________________________

________________________________________________________________

________________________________________________________________

End of Block: Symptoms Check and Health Impact
